# Genetic Regulation of Endothelial Vasomotor Function

**DOI:** 10.3389/fphys.2016.00571

**Published:** 2016-11-25

**Authors:** Seung Kyum Kim, Michael P. Massett

**Affiliations:** ^1^Department of Health and Kinesiology, Texas A&M UniversityCollege Station, TX, USA; ^2^Tufts Medical Center, Molecular Cardiology Research InstituteBoston, MA, USA

**Keywords:** flow-mediated dilation, endothelium-dependent vasodilator, heritability, polymorphism, association studies, rodent strain comparison

## Abstract

The endothelium plays an important role in the regulation of vasomotor tone and the maintenance of vascular integrity. Endothelial dysfunction, i.e., impaired endothelial dependent dilation, is a fundamental component of the pathogenesis of cardiovascular disease. Although endothelial dysfunction is associated with a number of cardiovascular disease risk factors, those risk factors are not the only determinants of endothelial dysfunction. Despite knowing many molecules involved in endothelial signaling pathways, the genetic contribution to endothelial function has yet to be fully elucidated. This mini-review summarizes current evidence supporting the genetic contribution to endothelial vasomotor function. Findings from population-based studies, association studies for candidate genes, and unbiased large genomic scale studies in humans and rodent models are discussed. A brief synopsis of the current studies addressing the genetic regulation of endothelial responses to exercise training is also included.

## Introduction

The endothelium is an important modulator of vascular function, sensing changes in hemodynamic forces and blood-borne signals, and responding by releasing vasoactive molecules (Hintze and Vatner, 1984; Pohl et al., 1986; Rubanyi et al., 1986; Sinoway et al., 1989; Koller and Kaley, 1991; Hecker et al., 1993). Clinically, endothelial dysfunction is characterized by a reduced response to infusion of endothelium-dependent vasodilators, such as acetylcholine (ACh; Nabel et al., 1990; Hasdai and Lerman, 1999), or impaired flow-mediated dilation (FMD). FMD measures vasodilation induced by reactive hyperemia after release of acute occlusion of the brachial artery (Celermajer et al., 1992). This acute increase in blood flow exerts shear forces on the vessel that stimulate endothelial cells to release primarily nitric oxide (NO) which subsequently relaxes vascular smooth muscle (Pohl et al., 1986; Rubanyi et al., 1986). Endothelial dysfunction is a predictor of future cardiovascular events (Yeboah et al., 2009; Inaba et al., 2010; Ras et al., 2013) and also contributes to the pathology of chronic diseases including diabetes (McVeigh et al., 1992; Williams et al., 1996), chronic kidney disease (Annuk et al., 2001; Stam et al., 2006), and Alzheimer's disease (Dede et al., 2007; Kelleher and Soiza, 2013).

Chan et al. estimated that known risk factors for coronary heart disease account for <20% of variation in vascular responses to vasodilator agents (Chan et al., 2001). Differences in resting blood flow may account for up to another 45%. Therefore, the remaining 30–40% of variation in vascular function is unexplained. Although one potential factor is genetic variation, genetic regulation of endothelial vasomotor function is poorly understood. This mini-review will concentrate on the role of genetic variants on vascular function, emphasizing endothelium-dependent responses. We will summarize the results from candidate gene studies in humans and rodents and highlight genome-wide studies of genetic regulation of endothelial function.

## Genetic regulation of endothelial function in humans

### Familial resemblance and heritability

In children and first-degree relatives of individuals with premature coronary disease, impaired endothelial function occurs before onset of overt disease and is significantly and independently correlated with family history of premature coronary disease (Clarkson et al., 1997; Gaeta et al., 2000). Furthermore, twin and family studies suggest endothelial function or FMD is a heritable trait. A significant familial aggregation was reported for FMD measured in 81 nuclear families with a heritability estimate of 0.58 for FMD in sibling pairs (Ryabikov et al., 2007). The Northern Manhattan Family Study reported heritability for FMD of 0.17 in Hispanic Caucasians after adjusting for age, sex, and cardiovascular disease (CVD) risk factors (Suzuki et al., 2008). Several twin studies have reported higher resemblance in FMD between monozygotic twins than dizygotic twins with heritability estimates between 0.24 and 0.44 (Jartti et al., 2002; Zhao et al., 2007; Hopkins et al., 2010). Two population-based studies estimated heritability for FMD to be 0.14–0.16 after accounting for confounding variables (Benjamin et al., 2004; Fisch et al., 2015). Collectively those findings support a role for genetics in development of endothelial dysfunction and heritability of endothelial function measured as FMD.

### Candidate gene studies

To date, the majority of research focusing on the genetic basis for endothelial vasomotor function has used the candidate gene approach. Association studies in humans have shown polymorphisms in genes related to vascular function [e.g., angiotensin converting enzyme, angiotensin II type 1 receptor, cytochrome b-245 alpha chain (*CYBA*), nitric oxide synthase 3 (*NOS3*), and GTP cyclohydrolase 1 (*GCH1*)] exhibit a range of effects on endothelial function (Table 1; Celermajer et al., 1994; Schächinger et al., 2001; Rossi et al., 2003; Schneider et al., 2003; Cattaruzza et al., 2004; Fricker et al., 2004; Paradossi et al., 2004; Demirel et al., 2005; Fan et al., 2007; Kiliszek et al., 2007; Antoniades et al., 2008; Ingelsson et al., 2008; Liao et al., 2010; Akpinar et al., 2014; Dong et al., 2014; Rafiq et al., 2014; Wolkow et al., 2014; Li et al., 2015). Two polymorphisms in *NOS3*, T^−786^ → C and G^894^ → T, are the most studied. T^−786^ → C resides in the promoter region of *NOS3* and regulates transcriptional initiation (Nakayama et al., 1999). The CC genotype at T^−786^ → C is associated with blunted forearm blood flow responses to ACh in hypertensive subjects (Rossi et al., 2003) and no increases in *NOS3* mRNA and endothelial nitric oxide synthase (eNOS) protein expression in response to laminar shear stress in endothelial cells from coronary heart patients (Cattaruzza et al., 2004). The G^894^ → T polymorphism in exon 7 of *NOS3* results in substitution of glutamate with aspartate at codon 298 (also denoted as Glu298Asp; Marsden et al., 1993). This polymorphism was significantly associated with FMD; TT genotype carriers had higher FMD than GG or GT genotype carriers (Ingelsson et al., 2008). However, these results are not consistent in the literature (Paradossi et al., 2004; Ingelsson et al., 2008). Moreover, in a cohort of 1446 subjects from the Framingham Heart Study, no significant associations were observed between FMD and 18 single nucleotide polymorphisms (SNPs) in *NOS3*, including T^−786^ → C and Glu298Asp (Kathiresan et al., 2005). Thus, the effects of *NOS3* polymorphisms on endothelial function are variable and might depend on the study population or other genetic or environmental factors.

**Table 1:** **Polymorphisms of candidate genes associated with human endothelial vasomotor function**.

**Study**	**Subject**	**Age (year)**	**Measurement**	**Gene (variant)**	**Genotype frequency (n)**	**Polymorphic effect**
Akpinar et al., 2014	255 healthy	35 ± 2	Baseline FMD	NOS3 (intron 4 a/b)	aa: 3/ab: 42/bb: 210	No effect
Demirel et al., 2005	129 hypertension	20–50	Baseline FMD	NOS3 (intron 4 a/b)	aa: 1/ab: 31/bb: 97	No effect
Cattaruzza et al., 2004	99 CHD	62 ± 1	Responses to ACh in SV	NOS3 (T^−786^ → C)	TT: 41/TC: 45/CC: 13	Vasorelaxation to ACh ↓ in CC genotype
Erbs et al., 2003	67 CAD		Intra-arterial infusion of ACh and response to ET	NOS3 (T^−786^ → C)	TT: 25/TC: 27/CC: 15	Baseline APV and response to ET ↓ in T allele carriers
Fricker et al., 2004	72 healthy male	27 ± 1	Intravenous infusion of BKN	NOS3 (T^−786^ → C)	TT: 27/TC: 32/CC: 13	No effect
Negrao et al., 2010	72 healthy male	18–35	Baseline FBF and response to ET	NOS3 (T^−786^ → C)	TT: 37/TC&CC: 35	FBF change during exercise ↓ in TT genotype, but no effect on FBF response to ET
Paradossi et al., 2004	118 healthy	21–45	Baseline FMD	NOS3 (T^−786^ → C)	TT: 43/TC: 58/CC: 17	No effect
Rossi et al., 2003	137 hypertension	49 ± 9	Baseline FBF	NOS3 (T^−786^ → C)	TT: 38/TC: 69/CC: 30	FBF ↑ in TT genotype
Erbs et al., 2003	67 CAD		Intra-arterial infusion of ACh and response to ET	NOS3 (G^894^ → T)	GG: 3/GT: 31/TT: 33	Baseline APV and response to ET ↓ in G allele carriers
Fricker et al., 2004	72 healthy male	27 ± 1	Intravenous infusion of BKN	NOS3 (G^894^ → T)	GG: 29/GT: 28/TT: 15	No effect
Kiliszek et al., 2007	44 CAD/CHD	40–75	Baseline FMD	NOS3 (G^894^ → T)	GG: 17/GT: 25/TT: 2	No effect
Paradossi et al., 2004	118 healthy	21–45	Baseline FMD	NOS3 (G^894^ → T)	GG: 43/GT: 57/TT: 18	FMD ↓ in TT genotype
Rossi et al., 2003	137 hypertension	49 ± 9	Baseline FBF	NOS3 (G^894^ → T)	GG: 39/GT: 51.9/TT: 9.1 (%)	No effect
Ingelsson et al., 2008	959 unspecified	Aged 70	Baseline FMD	NOS3 (23 SNPs)		G^894^ → T is related to FMD (FMD ↑ in TT genotype)
Ingelsson et al., 2008	959 unspecified	Aged 70	Intra-arterial infusion of ACh	NOS3 (23 SNPs)		Only 14th intron A/G is related to EDV (EDV ↑ in heterozygous genotype)
Kathiresan et al., 2005	1446 unspecified	62 ± 9	Baseline FMD	eNOS (18 SNPs)		4 variants (hCV33219467, rs1800783, rs1800781, rs1007311) are related to FMD only in men
Akpinar et al., 2014	255 healthy	35 ± 2	Baseline FMD	ACE (16th intron I/D)	II: 45/ID: 111/DD: 99	No effect
Celermajer et al., 1994	184 healthy	15–73	Baseline FMD	ACE (16th intron I/D)	II: 46/ID: 89/DD: 49	No effect
Demirel et al., 2005	129 hypertension	20–50	Baseline FMD	ACE (16th intron I/D)	II: 23/ID: 63/DD: 43	No effect
Akpinar et al., 2014	255 healthy	35 ± 2	Baseline FMD	AT1R (A^1166^ → C)	AA: 184/AC: 68/CC: 3	FMD ↓ in C allele carriers
Demirel et al., 2005	129 hypertension	20–50	Baseline FMD	AT1R (A^1166^ → C)	AA: 94/AC: 32/CC: 3	No effect
Kiliszek et al., 2007	44 CAD	40–75	Baseline FMD	AT1R (A^1166^ → C)	AA: 21/AC: 20/CC: 3	No effect
Li et al., 2015	483 MI	65–79	Baseline FMD	AT1R (A^1166^ → C)	AA: 216/AC: 155/CC: 112	FMD ↓ in C allele carriers
Fan et al., 2007	2058 unspecified	24–39	Baseline FMD	CYBA (C^242^ → T)	CC: 1362/CT: 616/TT: 80	FMD ↓ in CC genotype
Fricker et al., 2004	72 healthy male	27 ± 1	Intravenous infusion of BKN	CYBA (C^242^ → T)	CC: 32/CT: 31/TT: 9	No effect
Kiliszek et al., 2007	44 CAD/CHD	40–75	Baseline FMD	CYBA (C^242^ → T)	CC: 21/CT: 19/TT: 4	No effect
Rafiq et al., 2014	75 hypertension	30–65	Baseline FMD	CYBA (C^242^ → T)	CC: 35/CT: 38/TT: 2	FMD ↑ in T allele carriers
Schächinger et al., 2001	93 CAD and healthy	40s–60s	Baseline FMD	CYBA (C^242^ → T)	CC: 44/CT: 38/TT: 11	No effect on normal subject; but FMD ↓ in CC genotype of patients
Schneider et al., 2003	90 HC	46 ±14	Baseline FBF	CYBA (C^242^ → T)	CC: 32/CT: 47/TT: 11	No effect
Kiliszek et al., 2007	44 CAD/CHD	40–75	Baseline FMD	CYBA (A^640^ → G)	AA: 18/AG: 17/GG: 7	No effect
Antoniades et al., 2008	347 CAD	65 ± 1	Responses to ACh in SV	GCH1 (3 SNPs)	OO (non) /XO (het) /XX (homo)	Vasorelaxation to ACh ↓ in X haplotype carriers
Liao et al., 2010	611 T2DM	40s–60s	Baseline FMD	GCH1 (C^59038^ → T)	CC: 214/CT: 277/TT: 120	FMD ↓ in T allele carriers
Wolkow et al., 2014	182 T2DM	37–72	Baseline FMD	GCH1 (5 SNP_*S*_)		Only rs841 in the 3'-UTR is related to FMD (FMD ↓ in aa genotype)
Hovingh et al., 2004	201 healthy	0.8–90	Baseline FMD	APO A-I (L178P mutation)	L178P carriers: 54/noncarrier: 147	Log [FMD] ↓ in carriers
Guangda and Yuhua, 2003	255 T2DM	58 ± 8	Baseline FMD	APOE (e2, e3, e4)	e2/2e3/2: 34/e3/3: 161/e4/3e4/4: 58	FMD ↓ in e4 allele carriers
Guangda et al., 2006	144 T2DM	57 ± 5	Baseline FMD	APOE (e2, e3, e4)	e2/2e3/2: 24/e3/3: 90/e4/3e4/4: 30	FMD ↓ in e4 allele carriers
Irace et al., 2008	118 Diabetic male	56 ± 8	Baseline FMD	PON1 (Q^192^ → R)	QQ: 64/QR+RR: 54	FMD ↓ in R allele carriers
Alves et al., 2013	71 healthy male	19–36	FBF response to ET	BDKRB2 (exon1 +9/–9)	–9/–9: 17, –9/+9: 34, +9/+9: 20	FBF response to ET ↑ in –9/–9 genotype
Lemos et al., 2016	304 healthy male	19–36	FBF response to ET	BDNF (Val66Met)	Val66Val: 221/Val66Met: 83	FBF response to ET ↑ in Val66Val genotype
Park et al., 2007	47 pre-/stage I hypertension	50–75	FBF and response to ET	NFKB1 (I^−94^ → D)	II: 8/ID: 23/DD: 5	Hyperemic FBF ↓ in DD genotype and its response to ET ↑ in I allele carriers

*CHD, Coronary heart disease; CAD, Coronary artery disease; MI, Myocardial infarction; HC, Hypercholoesterolaemia; T2D, Type 2 Diabetes; SV, saphenous veins; ACh, Acetylcholine; BKN, Bradykinin; FMD, Flow-medicated dilation; APV, Average peak velocity; ET, Exercise training; BAD, Brachial artery diameter (mm); FBF, Forearm blood flow; EDV, Endothelium-dependent vasodilation; NOS3, Nitric oxide synthase 3; ACE, Angiotensin converting enzyme; AT1R, Angiotensin II receptor, type 1; CYBA, Cytochrome b-245 alpha chain; GCH1, GTP cyclohydrolase I; APO A-I, Apolipoprotein A-I; APOE, Apolipoprotein E; PON1, Paraoxonase 1; BDKRB2, Bradykinin receptor B_2_; BDNF, Brain-derived neurotrophic factor; NFKB1, Nuclear factor NF-kappa-B p105 subunit*.

Polymorphisms in genes regulating NO bioavailability also have been tested for associations with endothelial function. One is the C^242^ → T polymorphism located in exon 4 of the p22phox subunit of NADPH oxidase (*CYBA*), which decreases ${\text{O}}_{2}^{™}$ production. In >2000 subjects, Fan and colleagues found that T allele carriers showed higher brachial FMD (%) than C allele carriers (Fan et al., 2007). Schächinger also reported T allele carriers had greater endothelium-dependent vasodilator responses in epicardial arteries as compared to C allele carriers (Schächinger et al., 2001). However, the polymorphic effect of C^242^ → T on endothelial function is not consistently observed in smaller studies (Schneider et al., 2003; Fricker et al., 2004; Kiliszek et al., 2007; Rafiq et al., 2014). In contrast, polymorphisms in GTP cyclohydrolase 1 (*GCH1*), the rate-limiting enzyme in the synthesis of eNOS cofactor 6R-tetrahydrobiopterin (BH_4_), significantly affect endothelium-dependent vasodilation (Antoniades et al., 2008; Liao et al., 2010; Wolkow et al., 2014). Thus, variation in genes affecting NO bioavailability might have clinical implications for treating endothelial dysfunction. However, consistent association of any polymorphism with endothelial function is not yet firmly established.

In addition to genes affecting NO bioavailability and responsiveness, polymorphisms in other vascular biology related genes also are potential modifiers of endothelial vasomotor function. An apolipoprotein A-I mutation (L178P) was associated with impaired FMD and enhanced CVD risk (Hovingh et al., 2004). Similarly, FMD was lower in diabetic patients without angiopathy carrying the e4 allele of apolipoprotein E (*APOE*, ApoE4) (Guangda and Yuhua, 2003; Guangda et al., 2006). The recent finding that the R952Q variant in apolipoprotein E receptor 2 (*ApoER2*) and ApoE4 negatively affect NO-mediated endothelial cell repair and eNOS activation substantiated the link between ApoE4 and impaired endothelial function (Ulrich et al., 2014). Furthermore, polymorphisms in paraoxonase 1 (*PON1*), an antioxidant linked to high-density lipoproteins, have been associated with endothelial dysfunction. Male diabetics with the arginine allele of the Gln192Arg polymorphism in *PON1* had reduced FMD compared with male homozygotes for the glutamine allele (Irace et al., 2008). Conversely, variation in *PON1* was associated with coronary endothelial dysfunction in women, but not men (Yoshino et al., 2016). Those results suggest that sex, vessel size, and disease status might influence the variant effects of *PON1*. Further investigation into the role of this gene on endothelial function is warranted. Collectively, these associations suggest genetic regulation of endothelial vasomotor function involves pathways not directly associated with the canonical NO pathway.

### Genome wide association studies

Over the last decade, genome-wide association studies (GWAS) have been used to identify novel genetic loci underlying CVD and other chronic diseases, but GWAS for endothelial function have been limited. Vasan and colleagues conducted an association study for several cardiovascular traits including FMD (%) and hyperemic flow velocity in 1345 subjects from the Framingham Heart Study using a 100k SNP set (Vasan et al., 2007). They identified several SNPs associated with each trait, including SNPs in cystic fibrosis transmembrane conductance regulator (*CFTR*) and phosphodiesterase 5A (*PDE5A*). *CFTR* encodes for a chloride ion channel expressed in endothelial and vascular smooth muscle cells (Tousson et al., 1998; Robert et al., 2005). *PDE5A* encodes a cGMP-specific phosphodiesterase that regulates smooth muscle relaxation (Kass et al., 2007). Although those findings have not been replicated, that was the first GWAS to directly investigate endothelial function in a large size sample population, offering a fundamental framework for GWAS of endothelial function.

Subsequently, Yoshino and colleagues performed an association study for coronary vascular responses to ACh, an index of coronary endothelial function, in 643 female and male subjects (Yoshino et al., 2016). They utilized 1536 tag SNPs located in genes previously connected with cardiovascular physiology and pathology. Variants in adenosine A1 receptor (*ADORA1*) were associated with endothelial dysfunction in the entire cohort, whereas variants in adenosine A3 receptor (*ADORA3*) and lipoprotein(a) (*LPA*) had the strongest associations with increased risk of endothelial dysfunction in women only. Their sex-specific results further suggest genetic regulation of endothelial (dys)function might differ between sexes and future studies must consider sex by genotype interactions.

### Genetic contribution to endothelial responses to exercise training

Exercise training is a non-pharmacological means to improve endothelial function. However, there is growing acceptance that phenotypic responses to exercise training are heterogeneous. Green observed a wide range of inter-individual variation in FMD (%) changes after exercise training (Green et al., 2014). Among 182 subjects, 76% exhibited improved FMD, while 24% showed no changes or even decreased FMD after exercise training. Thus, exercise training can exert non-uniform effects on endothelial function among individuals. Hopkins (Hopkins et al., 2012) provided evidence for a genetic contribution to these variable responses. After 8 weeks of aerobic exercise training changes in FMD (%) were highly correlated in monozygotic twins (*r* = 0.63), whereas changes in FMD (%) were not correlated in dizygotic twins (*r* = 0.37). The estimated heritability of training-induced changes in FMD was 0.74, which is significantly higher than estimates of heritability for FMD in non-exercise training studies. Whether genetic influence on endothelial responses to exercise training is also significantly greater remains to be determined.

Because improvements in endothelial function with exercise training can occur primarily through changes in NO signaling, the impact of polymorphisms in *NOS3* and related genes on endothelial responses to exercise training has been examined. In coronary artery disease patients, exercise training for 4 weeks improved ACh-induced average peak velocity (APV) in coronary arteries relative to *NOS3* polymorphism (Erbs et al., 2003). Patients carrying C allele at T^−786^ → C had a smaller improvement in APV (~36%) than patients carrying T allele (~ 81%), whereas a polymorphic effect of G^894^ → T was not observed. Similarly, 18 weeks of exercise training increased forearm vascular conductance during handgrip exercise in TT carriers, but not in CT or CC carriers at T^−786^ → C of *NOS3* (Negrao et al., 2010). For other vascular-related genes, there is limited information regarding their role in improved endothelial function with exercise training (Park et al., 2007; Alves et al., 2013; Lemos et al., 2016). The mixed results from those and other association studies imply there is a complicated interaction between genetic factors and exercise on endothelial adaptation to exercise training.

## Genetic regulation of endothelial function in rodents

### Candidate gene studies

Candidate gene studies in rodents also have focused on genes previously linked to endothelial physiology and pathology. Many of these genes have been reviewed elsewhere (Faraci and Sigmund, 1999), and full discussion is beyond the scope of this review. Typically those studies have been conducted using knockout mouse models to test a direct functional role of a gene in a vascular phenotype or investigate associated-signaling pathways (Babinet, 2000; Hall et al., 2001). They also have been useful in identifying differences in endothelial function throughout the vascular tree (Gongora et al., 2006), but the clinical relevance of complete gene knockout or overexpression is unclear. Recently, mouse models mimicking subtle genetic variation seen in humans have been developed. A single point mutation at the S1176 phosphorylation site on eNOS that increased enzyme activity also improved endothelial function and clinical outcomes in mice (Atochin et al., 2007; Li et al., 2013). Conversely, responses to ACh are attenuated in blood vessels from mice carrying cell specific dominant-negative versions of PPARγ that mimic human mutations in this gene, but only after high fat diet or angiotensin II infusion (Beyer et al., 2008; Hu et al., 2016). Thus, in mice, genetic modifications resembling human polymorphisms or mutations provide proof of concept that small genetic changes can elicit relevant (patho)physiological changes in endothelial vasomotor function without eliciting marked systemic changes or deleterious effects due to complete gene loss or overexpression. These models can facilitate more precise mechanistic investigation of vascular function, but also provide information that is more translatable to human pathophysiology.

### Mouse and rat strain comparisons

An alternative approach for investigating the genetic basis for vascular function is an inter-strain comparison of vascular phenotypes among different inbred rodent strains. Phenotypic diversity across inbred strains permits identification of novel gene(s) responsible for a phenotype via association analysis between phenotype and genotype over the entire genome (Flaherty et al., 2005; Flint and Eskin, 2012). Although no large-scale (≥10) rodent strain surveys for vascular function have been published, several small studies indicate strain-dependent differences in endothelium-dependent vasorelaxation (Table 2; Bendall et al., 2002; Ryan et al., 2002; Chen et al., 2007). Each study reported impaired responses to ACh in isolated aorta from at least one mouse strain. However, responses to other endothelium-dependent agonists were not consistent, nor were responses in vessels from other vascular beds. Ryan found that two 129-substrains of mice had markedly reduced responses to ACh in isolated aorta, but not carotid arteries, compared with 5 other inbred mouse strains (Ryan et al., 2002). Alternatively, outbred MF1 mice showed impaired responses to ACh in isolated aorta and reduced coronary relaxation responses to bradykinin and ACh in isolated perfused hearts (Bendall et al., 2002). Thus, genetic background influences endothelial function in mice; however, the magnitude might vary depending on the vascular bed. In addition, parallel to lower responses to ACh in aorta from SJL mice, eNOS, and SOD-2 protein expression were lower (Chen et al., 2007). Similarly, impaired responses to ACh in aorta from outbred MF1 mice improved after incubation with the superoxide scavenger Tiron (Bendall et al., 2002). Together these findings imply decreased NO bioavailability may contribute to impaired vasorelaxation responses. However, mechanisms underlying variation in eNOS and antioxidant signaling pathway protein content among inbred mouse strains remain to be elucidated.

**Table 2:** **Strain-dependent differences in endothelial vasomotor function in animals**.

**Study**	**Species**	**Strain**	**Age (wk)**	**Sex**	**Vessel type**	**Vasoactive agents**	**Responses**
Ryan et al., 2002	Mouse	A/J, BALb/cJ, C57BL/6J, C3HeB/J, 129P3/J, 129X1/SvJ, and SWR/J	16 - 22	Male	Aorta	ACh	Compared to C57BL/6J, ↓ response in 129P3/J and 129X1/SvJ and ↑ response in A/J
Bendall et al., 2002	Mouse	MF1, 129sv and C57BL/6J	8 - 10	Male	Coronary vessel (Langendorff) and aorta	BK (coronary vessel) and ACh and A23187 (both vessels)	- ↓ response to both BK and ACh in coronary vessels from MF1 - ↓ response to both bradykinin and ACh in aortas from MF1
Chen et al., 2007	Mouse	C3HeB/FeJ, FVB/NJ and SJL/J	16 - 18	Both	Aorta	ACh	↓ response in aortas from SJL/J
Kunert et al., 2006	Rat	Consomic panel (Substitution of BN onto SS background)	10	Male	Aorta	ACh	Compared to SS parental strain, When fed a low salt (0.4% NaCl) diet, - ↑ sensitivity (EC_50_) in Chr. 16 and Y consomic strains - ↓ sensitivity in Chr. 9, 13, 20 consomic strains When fed a high salt (4.0% NaCl) diet, - ↑ sensitivity in Chr. 2 and 8 consomic strains - ↓ sensitivity in Chr. 4, 10, 13, 15, 19, 20, and X consomic strains
Kunert et al., 2008	Rat	Consomic panel (Substitution of BN onto SS background)	10	Female	Aorta	ACh	Compared to SS parental strain, When fed a low salt (0.4% NaCl) diet, - ↑ maximal relaxation to ACh in Chr. 2, 6, 7, 9, 13, 20 consomic strains When fed a high salt (4.0% NaCl) diet, - ↑ maximal relaxation to ACh in Chr. 5, 13 and 17 consomic strains
Kunert et al., 2010	Rat	Consomic panel (Substitution of BN onto FHH background)	10	Male	Aorta	ACh	Compared to FHH parental strain, When fed a low salt (0.4% NaCl) diet, - ↑ sensitivity in Chr. 3, 4, 5, 11, 12, 14, and Y consomic strains - ↑ sensitivity in Chr. 10 consomic strain - ↑ maximal relaxation to ACh in Chr. 5 consomic strain When fed a high salt (4.0% NaCl) diet, - ↑ sensitivity in Chr. 5 consomic strain - ↑ sensitivity in Chr. 3 consomic strain - ↑ maximal relaxation to ACh in Chr. 6 consomic strain - ↑ maximal relaxation to ACh in Chr. 1 consomic strain
Kunert et al., 2010	Rat	Consomic panel (Substitution of BN onto FHH background)	10	Female	Aorta	ACh	Compared to FHH parental strain, When fed a low salt (0.4% NaCl) diet, - ↑ maximal relaxation to ACh in Chr. 5, 15 and 17 consomic strains When fed a high salt (4.0% NaCl) diet, - ↑ maximal relaxation to ACh in Chr. X consomic strain

*ACh, Acetylcholine; BK, Bradykinin; BN, Brown Norway rat strain; SS, Dahl salt sensitive rat strain; FHH, Fawn hooded hypertensive rat strain; Chr., Chromosome*.

Supportive evidence also has been derived from investigations in genetically manipulated rats. A consomic rat panel was created by substituting chromosomes from normotensive Brown Norway (BN) rats onto the background of the Dahl salt sensitive (SS) inbred rat strain (Cowley, 2003). Phenotype comparisons across consomic and inbred parental SS strains afford opportunity to discover chromosomes containing genes contributing to the phenotype of interest. Using that rat consomic panel, aortic rings from strains with substituted chromosomes 16 and Y had greater ACh sensitivity, while aortic rings from strains carrying chromosomes 9, 13, and 20 had reduced sensitivity compared with aorta from inbred parental SS rats (Kunert et al., 2006). These results indicate that chromosomes 9, 13, 16, 20, and Y contain gene(s) responsible for ACh sensitivity. These investigators later utilized a consomic rat panel constructed from BN and Fawn Hooded Hypertensive (FHH) rat strains (Kunert et al., 2010). ACh sensitivity differed for consomic rats of chromosomes 3, 4, 5, 10, 11, 12, 14, and Y compared to parental FHH inbred rats. Only the Y chromosome was identified in both studies as influencing ACh sensitivity, implying chromosomes responsible for endothelial sensitivity to ACh are strain-specific in rats (Kunert et al., 2006, 2010). Collectively, results from animal studies clearly indicate endothelial function has genetic regulation and support comprehensive genomic scans via objective and unbiased hypothesis-free tests to identify novel genomic loci responsible for regulating endothelial function.

## Future directions

The endothelium has a critical role in maintaining vascular integrity and protecting against cardiovascular disease. Accumulated data indicate endothelial function is a heritable trait regulated by polygenic factors; however, these genetic factors have not been fully elucidated. Given that single genetic variants generally have only small to modest functional effects, future studies should focus on endothelial function in larger populations (humans or rodents) to facilitate genome wide studies and comprehensively unravel the complex genetic basis of endothelial function. In addition, the majority of studies cited here utilized FMD, a measure of endothelial function in a conduit artery. However, many cardiovascular diseases are associated with endothelial dysfunction in resistance vessels. Collectively, there is less evidence regarding genetic regulation of endothelial function in small vessels and FMD measurements do not always agree with assessment of resistance artery endothelial function via infusion of ACh (Lind et al., 2011). Therefore, studies directly assessing endothelial function in resistance arteries are needed to better understand the genetic regulation of resistance artery endothelial function and its contribution to cardiovascular disease progression.

Because many GWAS identify SNPs outside protein coding regions or in non-coding intervals, the contribution of small non-coding RNA (e.g., lncRNA, microRNA) in modulating endothelial function should be addressed. Emerging evidence suggests microRNA levels are associated with impaired responses to ACh in humans (Widmer et al., 2014) and rodents (Norata et al., 2012; Li et al., 2016). Heritable changes in gene activity and expression also can be the result of epigenetic changes. Recent evidence suggests epigenetic changes such as those induced by histone methyltransferase Set7 are associated with endothelial dysfunction, including impaired FMD in diabetics (Paneni et al., 2015). Furthermore, responses to ACh were impaired in aortic segments from heterozygous lysine-specific demethylase-1 (LSD-1) mice (Pojoga et al., 2011). A polymorphism in LSD-1, which induces histone H3 demethylation, is associated with salt-sensitive hypertension in humans (Williams et al., 2012). Therefore, expanding the search for genetic regulators of endothelial vasomotor tone beyond candidate gene studies could facilitate discovery of modulators of endothelial function and cardiovascular disease.

## Author contributions

SK drafted the manuscript. SK and MM revised the manuscript.

## Funding

This work was supported by grant R01 HL085918 (to MM) from the National Institutes of Health.

### Conflict of interest statement

The authors declare that the research was conducted in the absence of any commercial or financial relationships that could be construed as a potential conflict of interest.

## References

[B1] Akpinar T. S. Ozkok A. Kose M. Atas R. Sumnu A. Bakkaloglu O. K.. (2014). Endothelial constitutive nitric oxide synthase, angiotensin converting enzyme, angiotensin II type 1 receptor gene polymorphisms and endothelial functions in healthy individuals. Eur. Rev. Med. Pharmacol. Sci. 18, 39–45. Available online at: http://www.europeanreview.org/article/6452 24452940

[B2] Alves C. R. Alves G. B. Pereira A. C. Trombetta I. C. Dias R. G. Mota G. F.. (2013). Vascular reactivity and ACE activity response to exercise training are modulated by the +9/-9 bradykinin B(2) receptor gene functional polymorphism. Physiol. Genomics 45, 487–492. 10.1152/physiolgenomics.00065.2012 23613132

[B3] Annuk M. Lind L. Linde T. Fellstrom B. (2001). Impaired endothelium-dependent vasodilatation in renal failure in humans. Nephrol. Dial. Transplant 16, 302–306. 10.1093/ndt/16.2.302 11158404

[B4] Antoniades C. Shirodaria C. Van Assche T. Cunnington C. Tegeder I. Lotsch J.. (2008). GCH1 haplotype determines vascular and plasma biopterin availability in coronary artery disease effects on vascular superoxide production and endothelial function. J. Am. Coll. Cardiol. 52, 158–165. 10.1016/j.jacc.2007.12.062 18598896 PMC2699614

[B5] Atochin D. N. Wang A. Liu V. W. Critchlow J. D. Dantas A. P. Looft-Wilson R.. (2007). The phosphorylation state of eNOS modulates vascular reactivity and outcome of cerebral ischemia *in vivo*. J. Clin. Invest. 117, 1961–1967. 10.1172/JCI29877 17557122 PMC1884686

[B6] Babinet C. (2000). Transgenic mice: an irreplaceable tool for the study of mammalian development and biology. J. Am. Soc. Nephrol. 11 Suppl 16, S88–94. Available online at: http://jasn.asnjournals.org/content/11/suppl_2/S88.long 11065337

[B7] Bendall J. K. Heymes C. Wright T. J. Wheatcroft S. Grieve D. J. Shah A. M.. (2002). Strain-dependent variation in vascular responses to nitric oxide in the isolated murine heart. J. Mol. Cell. Cardiol. 34, 1325–1333. 10.1006/jmcc.2002.2083 12392993

[B8] Benjamin E. J. Larson M. G. Keyes M. J. Mitchell G. F. Vasan R. S. Keaney J. F.. (2004). Clinical correlates and heritability of flow-mediated dilation in the community: the Framingham Heart Study. Circulation 109, 613–619. 10.1161/01.CIR.0000112565.60887.1E 14769683

[B9] Beyer A. M. de Lange W. J. Halabi C. M. Modrick M. L. Keen H. L. Faraci F. M.. (2008). Endothelium-specific interference with peroxisome proliferator activated receptor gamma causes cerebral vascular dysfunction in response to a high-fat diet. Circ. Res. 103, 654–661. 10.1161/CIRCRESAHA.108.176339 18676352 PMC2583077

[B10] Cattaruzza M. Guzik T. J. Slodowski W. Pelvan A. Becker J. Halle M.. (2004). Shear stress insensitivity of endothelial nitric oxide synthase expression as a genetic risk factor for coronary heart disease. Circ. Res. 95, 841–847. 10.1161/01.RES.0000145359.47708.2f 15375006

[B11] Celermajer D. S. Sorensen K. E. Barley J. Jeffrey S. Carter N. Deanfield J. (1994). Angiotensin-converting enzyme genotype is not associated with endothelial dysfunction in subjects without other coronary risk factors. Atherosclerosis 111, 121–126. 10.1016/0021-9150(94)90197-X 7840807

[B12] Celermajer D. S. Sorensen K. E. Gooch V. M. Spiegelhalter D. J. Miller O. I. Sullivan I. D.. (1992). Non-invasive detection of endothelial dysfunction in children and adults at risk of atherosclerosis. Lancet 340, 1111–1115. 10.1016/0140-6736(92)93147-F 1359209

[B13] Chan N. N. Colhoun H. M. Vallance P. (2001). Cardiovascular risk factors as determinants of endothelium-dependent and endothelium-independent vascular reactivity in the general population. J. Am. Coll. Cardiol. 38, 1814–1820. 10.1016/S0735-1097(01)01669-2 11738279

[B14] Chen C. Korshunov V. A. Massett M. P. Yan C. Berk B. C. (2007). Impaired vasorelaxation in inbred mice is associated with alterations in both nitric oxide and super oxide pathways. J. Vasc. Res. 44, 504–512. 10.1159/000106751 17664889

[B15] Clarkson P. Celermajer D. S. Powe A. J. Donald A. E. Henry R. M. Deanfield J. E. (1997). Endothelium-dependent dilatation is impaired in young healthy subjects with a family history of premature coronary disease. Circulation 96, 3378–3383. 10.1161/01.CIR.96.10.3378 9396430

[B16] Cowley A. W. Jr. (2003). Genomics and homeostasis. Am. J. Physiol. Regul. Integr. Comp. Physiol. 284, R611–R627. 10.1152/ajpregu.00567.2002 12571070

[B17] Dede D. S. Yavuz B. Yavuz B. B. Cankurtaran M. Halil M. Ulger Z.. (2007). Assessment of endothelial function in Alzheimer's disease: is Alzheimer's disease a vascular disease? J. Am. Geriatr. Soc. 55, 1613–1617. 10.1111/j.1532-5415.2007.01378.x 17711428

[B18] Demirel S. Akkaya V. Cine N. Oflaz H. Yekeler E. Ozturk S.. (2005). Genetic polymorphisms and endothelial dysfunction in patients with essential hypertension: a cross-sectional case-control study. Neth. Heart J. 13, 126–131. 25696471 PMC2497295

[B19] Dong X. Li D. Liu H. Zhao Y. (2014). SOD3 and eNOS genotypes are associated with SOD activity and NOx. Exp. Ther. Med. 8, 328–334. 10.3892/etm.2014.1720 24944642 PMC4061193

[B20] Erbs S. Baither Y. Linke A. Adams V. Shu Y. Lenk K.. (2003). Promoter but not exon 7 polymorphism of endothelial nitric oxide synthase affects training-induced correction of endothelial dysfunction. Arterioscler. Thromb. Vasc. Biol. 23, 1814–1819. 10.1161/01.ATV.0000090128.11465.18 12907461

[B21] Fan M. Raitakari O. T. Kahonen M. Juonala M. Hutri-Kahonen N. Marniemi J.. (2007). CYBA C242T gene polymorphism and flow-mediated vasodilation in a population of young adults: the Cardiovascular Risk in Young Finns Study. J. Hypertens. 25, 1381–1387. 10.1097/HJH.0b013e32810bfe58 17563559

[B22] Faraci F. M. Sigmund C. D. (1999). Vascular biology in genetically altered mice: smaller vessels, bigger insight. Circ. Res. 85, 1214–1225. 10.1161/01.RES.85.12.1214 10590250

[B23] Fisch A. S. Yerges-Armstrong L. M. Backman J. D. Wang H. Donnelly P. Ryan K. A.. (2015). Genetic variation in the platelet endothelial aggregation receptor 1 gene results in endothelial dysfunction. PLoS ONE 10:e0138795. 10.1371/journal.pone.0138795 26406321 PMC4583223

[B24] Flaherty L. Herron B. Symula D. (2005). Genomics of the future: identification of quantitative trait loci in the mouse. Genome Res. 15, 1741–1745. 10.1101/gr.3841405 16339372

[B25] Flint J. Eskin E. (2012). Genome-wide association studies in mice. Nat. Rev. Genet. 13, 807–817. 10.1038/nrg3335 23044826 PMC3625632

[B26] Fricker R. Hesse C. Weiss J. Tayrouz Y. Hoffmann M. M. Unnebrink K.. (2004). Endothelial venodilator response in carriers of genetic polymorphisms involved in NO synthesis and degradation. Br. J. Clin. Pharmacol. 58, 169–177. 10.1111/j.1365-2125.2004.02130.x 15255799 PMC1884579

[B27] Gaeta G. De Michele M. Cuomo S. Guarini P. Foglia M. C. Bond M. G.. (2000). Arterial abnormalities in the offspring of patients with premature myocardial infarction. N.Engl. J. Med. 343, 840–846. 10.1056/NEJM200009213431203 10995863

[B28] Gongora M. C. Qin Z. Laude K. Kim H. W. McCann L. Folz J. R.. (2006). Role of extracellular superoxide dismutase in hypertension. Hypertension 48, 473–481. 10.1161/01.HYP.0000235682.47673.ab 16864745

[B29] Green D. J. Eijsvogels T. Bouts Y. M. Maiorana A. J. Naylor L. H. Scholten R. R.. (2014). Exercise training and artery function in humans: nonresponse and its relationship to cardiovascular risk factors. J. Appl. Physiol. (1985) 117, 345–352. 10.1152/japplphysiol.00354.2014 24947027

[B30] Guangda X. Linshuang Z. Jie H. Ling Y. Huijuan X. (2006). Apo e4 allele is associated with endothelium-dependent arterial dilation in women with type 2 diabetes. Diabetes Res. Clin. Pract. 72, 155–161. 10.1016/j.diabres.2005.10.004 16337304

[B31] Guangda X. Yuhua W. (2003). Apolipoprotein e4 allele and endothelium-dependent arterial dilation in Type 2 diabetes mellitus without angiopathy. Diabetologia 46, 514–519. 10.1007/s00125-003-1060-5 12739024

[B32] Hall B. Limaye A. Kulkarni A. B. (2001). Overview: Generation of Gene Knockout Mice. Hoboken, NJ: John Wiley & Sons, Inc. 10.1002/0471143030.cb1912s44 PMC2782548 19731224

[B33] Hasdai D. Lerman A. (1999). The assessment of endothelial function in the cardiac catheterization laboratory in patients with risk factors for atherosclerotic coronary artery disease. Herz 24, 544–547. 10.1007/BF03044226 10609161

[B34] Hecker M. Mulsch A. Bassenge E. Busse R. (1993). Vasoconstriction and increased flow: two principal mechanisms of shear stress-dependent endothelial autacoid release. Am. J. Physiol. 265(3 Pt 2), H828–H833. 8105699 10.1152/ajpheart.1993.265.3.H828

[B35] Hintze T. H. Vatner S. F. (1984). Reactive dilation of large coronary arteries in conscious dogs. Circ. Res. 54, 50–57. 10.1161/01.RES.54.1.50 6692499

[B36] Hopkins N. D. Stratton G. Cable N. T. Tinken T. M. Graves L. E. Green D. J. (2012). Impact of exercise training on endothelial function and body composition in young people: a study of mono- and di-zygotic twins. Eur. J. Appl. Physiol. 112, 421–427. 10.1007/s00421-011-1993-1 21573774

[B37] Hopkins N. Stratton G. Maia J. Tinken T. M. Graves L. E. Cable T. N.. (2010). Heritability of arterial function, fitness, and physical activity in youth: a study of monozygotic and dizygotic twins. J. Pediatr. 157, 943–948. 10.1016/j.jpeds.2010.06.005 20638076

[B38] Hovingh G. K. Brownlie A. Bisoendial R. J. Dube M. P. Levels J. H. Petersen W.. (2004). A novel apoA-I mutation (L178P) leads to endothelial dysfunction, increased arterial wall thickness, and premature coronary artery disease. J. Am. Coll. Cardiol. 44, 1429–1435. 10.1016/j.jacc.2004.06.070 15464323

[B39] Hu C. Lu K. T. Mukohda M. Davis D. R. Faraci F. M. Sigmund C. D. (2016). Interference with PPARgamma in endothelium accelerates angiotensin II-induced endothelial dysfunction. Physiol. Genomics 48, 124–134. 10.1152/physiolgenomics.00087.2015 26534936 PMC4729699

[B40] Inaba Y. Chen J. A. Bergmann S. R. (2010). Prediction of future cardiovascular outcomes by flow-mediated vasodilatation of brachial artery: a meta-analysis. Int. J. Cardiovasc. Imaging 26, 631–640. 10.1007/s10554-010-9616-1 20339920

[B41] Ingelsson E. Syvänen A. C. Lind L. (2008). Endothelium-dependent vasodilation in conduit and resistance vessels in relation to the endothelial nitric oxide synthase gene. J. Hum. Hypertens. 22, 569–578. 10.1038/jhh.2008.37 18463668

[B42] Irace C. Cortese C. Fiaschi E. Scavelli F. Liberatoscioli L. Federici G.. (2008). The influence of PON1 192 polymorphism on endothelial function in diabetic subjects with or without hypertension. Hypertens. Res. 31, 507–513. 10.1291/hypres.31.507 18497471

[B43] Jartti L. Rönnemaa T. Kaprio J. Järvisalo M. J. Toikka J. O. Marniemi J.. (2002). Population-based twin study of the effects of migration from Finland to Sweden on endothelial function and intima-media thickness. Arterioscler. Thromb. Vasc. Biol. 22, 832–837. 10.1161/01.ATV.0000013313.70875.A7 12006398

[B44] Kass D. A. Champion H. C. Beavo J. A. (2007). Phosphodiesterase type 5: expanding roles in cardiovascular regulation. Circ. Res. 101, 1084–1095. 10.1161/CIRCRESAHA.107.162511 18040025

[B45] Kathiresan S. Larson M. G. Vasan R. S. Guo C. Y. Vita J. A. Mitchell G. F.. (2005). Common genetic variation at the endothelial nitric oxide synthase locus and relations to brachial artery vasodilator function in the community. Circulation 112, 1419–1427. 10.1161/CIRCULATIONAHA.105.544619 16129794

[B46] Kelleher R. J. Soiza R. L. (2013). Evidence of endothelial dysfunction in the development of Alzheimer's disease: is Alzheimer's a vascular disorder? Am. J. Cardiovasc. Dis. 3, 197–226. 24224133 PMC3819581

[B47] Kiliszek M. Burzynska B. Styczynski G. Maciag M. Rabczenko D. Opolski G. (2007). A1166C polymorphism of the angiotensin AT1 receptor (AT1R) gene alters endothelial response to statin treatment. Clin. Chem. Lab. Med. 45, 839–842. 10.1515/CCLM.2007.151 17617024

[B48] Koller A. Kaley G. (1991). Endothelial regulation of wall shear stress and blood flow in skeletal muscle microcirculation. Am. J. Physiol. 260(3 Pt 2), H862–H868. 2000980 10.1152/ajpheart.1991.260.3.H862

[B49] Kunert M. P. Drenjancevic-Peric I. Dwinell M. R. Lombard J. H. Cowley A. W. Jr. Greene A. S.. (2006). Consomic strategies to localize genomic regions related to vascular reactivity in the Dahl salt-sensitive rat. Physiol. Genomics 26, 218–225. 10.1152/physiolgenomics.00004.2006 16772359

[B50] Kunert M. P. Dwinell M. R. Drenjancevic Peric I. Lombard J. H. (2008). Sex-specific differences in chromosome-dependent regulation of vascular reactivity in female consomic rat strains from a SSxBN cross. Am. J. Physiol. Regul. Integr. Comp. Physiol. 295, R516–R527. 10.1152/ajpregu.00038.2008 18509103 PMC2519925

[B51] Kunert M. P. Dwinell M. R. Lombard J. H. (2010). Vascular responses in aortic rings of a consomic rat panel derived from the Fawn Hooded Hypertensive strain. Physiol. Genomics 42A, 244–258. 10.1152/physiolgenomics.00124.2010 20841496 PMC3008365

[B52] Lemos J. R. Jr. Alves C. R. de Souza S. B. Marsiglia J. D. Silva M. S. Pereira A. C.. (2016). Peripheral vascular reactivity and serum BDNF responses to aerobic training are impaired by the BDNF Val66Met polymorphism. Physiol. Genomics 48, 116–123. 10.1152/physiolgenomics.00086.2015 26603150

[B53] Li P. Yin Y. L. Guo T. Sun X. Y. Ma H. Zhu M. L.. (2016). Inhibition of aberrant microRNA-133a expression in endothelial cells by statin prevents endothelial dysfunction by targeting GTP cyclohydrolase 1 *in vivo*. Circulation. [Epub ahead of print]. 10.1161/CIRCULATIONAHA.116.017949 27765794 PMC5120771

[B54] Li Q. Atochin D. Kashiwagi S. Earle J. Wang A. Mandeville E.. (2013). Deficient eNOS phosphorylation is a mechanism for diabetic vascular dysfunction contributing to increased stroke size. Stroke 44, 3183–3188. 10.1161/STROKEAHA.113.002073 23988642 PMC3864831

[B55] Li Y. Chen F. Zhang X. Gao Y. Wu C. Li H.. (2015). Angiotensin type 1 receptor A1166C gene polymorphism is associated with endothelial dysfunction and in-stent restenosis after percutaneous coronary intervention. Int. J. Clin. Exp. Pathol. 8, 7350–7357. 26261635 PMC4525969

[B56] Liao Y. F. Zeng T. S. Chen L. L. Li Y. M. Yu F. Hu L. J.. (2010). Association of a functional polymorphism (C59038T) in GTP cyclohydrolase 1 gene and Type 2 diabetic macrovascular disease in the Chinese population. J. Diabetes Complicat. 24, 313–319. 10.1016/j.jdiacomp.2009.04.003 19515581

[B57] Lind L. Berglund L. Larsson A. Sundstrom J. (2011). Endothelial function in resistance and conduit arteries and 5-year risk of cardiovascular disease. Circulation 123, 1545–1551. 10.1161/CIRCULATIONAHA.110.984047 21444885

[B58] Marsden P. A. Heng H. H. Scherer S. W. Stewart R. J. Hall A. V. Shi X. M.. (1993). Structure and chromosomal localization of the human constitutive endothelial nitric oxide synthase gene. J. Biol. Chem. 268, 17478–17488. 7688726

[B59] McVeigh G. E. Brennan G. M. Johnston G. D. McDermott B. J. McGrath L. T. Henry W. R.. (1992). Impaired endothelium-dependent and independent vasodilation in patients with type 2 (non-insulin-dependent) diabetes mellitus. Diabetologia 35, 771–776. 1511805 10.1007/BF00429099

[B60] Nabel E. G. Selwyn A. P. Ganz P. (1990). Large coronary arteries in humans are responsive to changing blood flow: an endothelium-dependent mechanism that fails in patients with atherosclerosis. J. Am. Coll. Cardiol. 16, 349–356. 10.1016/0735-1097(90)90584-C 2115539

[B61] Nakayama M. Yasue H. Yoshimura M. Shimasaki Y. Kugiyama K. Ogawa H.. (1999). T-786–>C mutation in the 5′-flanking region of the endothelial nitric oxide synthase gene is associated with coronary spasm. Circulation 99, 2864–2870. 10.1161/01.CIR.99.22.2864 10359729

[B62] Negrao M. V. Alves C. R. Alves G. B. Pereira A. C. Dias R. G. Laterza M. C.. (2010). Exercise training improves muscle vasodilatation in individuals with T786C polymorphism of endothelial nitric oxide synthase gene. Physiol. Genomics 42A, 71–77. 10.1152/physiolgenomics.00145.2009 20605946

[B63] Norata G. D. Pinna C. Zappella F. Elia L. Sala A. Condorelli G.. (2012). MicroRNA 143-145 deficiency impairs vascular function. Int. J. Immunopathol. Pharmacol. 25, 467–474. 10.1177/039463201202500216 22697078

[B64] Paneni F. Costantino S. Battista R. Castello L. Capretti G. Chiandotto S.. (2015). Adverse epigenetic signatures by histone methyltransferase Set7 contribute to vascular dysfunction in patients with type 2 diabetes mellitus. Circ. Cardiovasc. Genet. 8, 150–158. 10.1161/CIRCGENETICS.114.000671 25472959

[B65] Paradossi U. Ciofini E. Clerico A. Botto N. Biagini A. Colombo M. G. (2004). Endothelial function and carotid intima-media thickness in young healthy subjects among endothelial nitric oxide synthase Glu298–>Asp and T-786–>C polymorphisms. Stroke 35, 1305–1309. 10.1161/01.STR.0000126482.86708.37 15073390

[B66] Park J. Y. Farrance I. K. Fenty N. M. Hagberg J. M. Roth S. M. Mosser D. M.. (2007). NFKB1 promoter variation implicates shear-induced NOS3 gene expression and endothelial function in prehypertensives and stage I hypertensives. Am. J. Physiol. Heart Circ. Physiol. 293, H2320–H2327. 10.1152/ajpheart.00186.2007 17644577 PMC2614625

[B67] Pohl U. Holtz J. Busse R. Bassenge E. (1986). Crucial role of endothelium in the vasodilator response to increased flow *in vivo*. Hypertension 8, 37–44. 10.1161/01.HYP.8.1.37 3080370

[B68] Pojoga L. H. Williams J. S. Yao T. M. Kumar A. Raffetto J. D. do Nascimento G. R.. (2011). Histone demethylase LSD1 deficiency during high-salt diet is associated with enhanced vascular contraction, altered NO-cGMP relaxation pathway, and hypertension. Am. J. Physiol. Heart Circ. Physiol. 301, H1862–H1871. 10.1152/ajpheart.00513.2011 21873498 PMC3213961

[B69] Rafiq A. Aslam K. Malik R. Afroze D. (2014). C242T polymorphism of the NADPH oxidase p22PHOX gene and its association with endothelial dysfunction in asymptomatic individuals with essential systemic hypertension. Mol. Med. Rep. 9, 1857–1862. 10.3892/mmr.2014.1992 24573492

[B70] Ras R. T. Streppel M. T. Draijer R. Zock P. L. (2013). Flow-mediated dilation and cardiovascular risk prediction: a systematic review with meta-analysis. Int. J. Cardiol. 168, 344–351. 10.1016/j.ijcard.2012.09.047 23041097

[B71] Robert R. Norez C. Becq F. (2005). Disruption of CFTR chloride channel alters mechanical properties and cAMP-dependent Cl- transport of mouse aortic smooth muscle cells. J. Physiol. 568(Pt 2), 483–495. 10.1113/jphysiol.2005.085019 16081479 PMC1474747

[B72] Rossi G. P. Taddei S. Virdis A. Cavallin M. Ghiadoni L. Favilla S.. (2003). The T-786C and Glu298Asp polymorphisms of the endothelial nitric oxide gene affect the forearm blood flow responses of Caucasian hypertensive patients. J. Am. Coll. Cardiol. 41, 938–945. 10.1016/S0735-1097(02)03011-5 12651037

[B73] Rubanyi G. M. Romero J. C. Vanhoutte P. M. (1986). Flow-induced release of endothelium-derived relaxing factor. Am. J. Physiol. 250(6 Pt 2), H1145–H1149. 3487253 10.1152/ajpheart.1986.250.6.H1145

[B74] Ryabikov A. Malyutina S. Ryabikov M. Kuznetsova T. Staessen J. A. Nikitin Y. (2007). Intrafamilial correlations of carotid intima-media thickness and flow-mediated dilation in a Siberian population. Am. J. Hypertens. 20, 248–254. 10.1016/j.amjhyper.2006.09.005 17324734

[B75] Ryan M. J. Didion S. P. Davis D. R. Faraci F. M. Sigmund C. D. (2002). Endothelial dysfunction and blood pressure variability in selected inbred mouse strains. Arterioscler. Thromb. Vasc. Biol. 22, 42–48. 10.1161/hq0102.101098 11788459

[B76] Schächinger V. Britten M. B. Dimmeler S. Zeiher A. M. (2001). NADH/NADPH oxidase p22 phox gene polymorphism is associated with improved coronary endothelial vasodilator function. Eur. Heart J. 22, 96–101. 10.1053/euhj.2000.2123 11133215

[B77] Schneider M. P. Hilgers K. F. Huang Y. Delles C. John S. Oehmer S.. (2003). The C242T p22phox polymorphism and endothelium-dependent vasodilation in subjects with hypercholesterolaemia. Clin. Sci. 105, 97–103. 10.1042/CS20030003 12639216

[B78] Sinoway L. I. Hendrickson C. Davidson W. R. Jr. Prophet S. Zelis R. (1989). Characteristics of flow-mediated brachial artery vasodilation in human subjects. Circ. Res. 64, 32–42. 10.1161/01.RES.64.1.32 2642398

[B79] Stam F. van Guldener C. Becker A. Dekker J. M. Heine R. J. Bouter L. M.. (2006). Endothelial dysfunction contributes to renal function-associated cardiovascular mortality in a population with mild renal insufficiency: the Hoorn study. J. Am. Soc. Nephrol. 17, 537–545. 10.1681/ASN.2005080834 16382015

[B80] Suzuki K. Juo S. H. Rundek T. Boden-Albala B. Disla N. Liu R.. (2008). Genetic contribution to brachial artery flow-mediated dilation: the Northern Manhattan Family Study. Atherosclerosis 197, 212–216. 10.1016/j.atherosclerosis.2007.03.023 17462653 PMC2268620

[B81] Tousson A. Van Tine B. A. Naren A. P. Shaw G. M. Schwiebert L. M. (1998). Characterization of CFTR expression and chloride channel activity in human endothelia. Am. J. Physiol. 275(6 Pt 1), C1555–C1564. 9843717 10.1152/ajpcell.1998.275.6.C1555

[B82] Ulrich V. Konaniah E. S. Herz J. Gerard R. D. Jung E. Yuhanna I. S.. (2014). Genetic variants of ApoE and ApoER2 differentially modulate endothelial function. Proc. Natl. Acad. Sci. U.S.A. 111, 13493–13498. 10.1073/pnas.1402106111 25197062 PMC4169937

[B83] Vasan R. S. Larson M. G. Aragam J. Wang T. J. Mitchell G. F. Kathiresan S.. (2007). Genome-wide association of echocardiographic dimensions, brachial artery endothelial function and treadmill exercise responses in the Framingham Heart Study. BMC Med. Genet. 8:1. 10.1186/1471-2156-8-1 17903301 PMC1995617

[B84] Widmer R. J. Chung W. Y. Herrmann J. Jordan K. L. Lerman L. O. Lerman A. (2014). The association between circulating microRNA levels and coronary endothelial function. PLoS ONE 9:e109650. 10.1371/journal.pone.0109650 25310838 PMC4195668

[B85] Williams J. S. Chamarthi B. Goodarzi M. O. Pojoga L. H. Sun B. Garza A. E.. (2012). Lysine-specific demethylase 1: an epigenetic regulator of salt-sensitive hypertension. Am. J. Hypertens. 25, 812–817. 10.1038/ajh.2012.43 22534796 PMC3721725

[B86] Williams S. B. Cusco J. A. Roddy M. A. Johnstone M. T. Creager M. A. (1996). Impaired nitric oxide-mediated vasodilation in patients with non-insulin-dependent diabetes mellitus. J. Am. Coll. Cardiol. 27, 567–574. 10.1016/0735-1097(95)00522-6 8606266

[B87] Wolkow P. P. Kosiniak-Kamysz W. Osmenda G. Wilk G. Bujak-Gizycka B. Ignacak A.. (2014). GTP cyclohydrolase I gene polymorphisms are associated with endothelial dysfunction and oxidative stress in patients with type 2 diabetes mellitus. PLoS ONE 9:e108587. 10.1371/journal.pone.0108587 25369080 PMC4219671

[B88] Yeboah J. Folsom A. R. Burke G. L. Johnson C. Polak J. F. Post W.. (2009). Predictive value of brachial flow-mediated dilation for incident cardiovascular events in a population-based study: the multi-ethnic study of atherosclerosis. Circulation 120, 502–509. 10.1161/CIRCULATIONAHA.109.864801 19635967 PMC2740975

[B89] Yoshino S. Cilluffo R. Prasad M. Best P. J. Atkinson E. J. Aoki T.. (2016). Sex-specific genetic variants are associated with coronary endothelial dysfunction. J. Am. Heart Assoc. 5:e002544. 10.1161/JAHA.115.002544 27091178 PMC4859270

[B90] Zhao J. Cheema F. A. Reddy U. Bremner J. D. Su S. Goldberg J.. (2007). Heritability of flow-mediated dilation: a twin study. J. Thromb. Haemost. 5, 2386–2392. 10.1111/j.1538-7836.2007.02760.x 17848176 PMC3113515

